# Autosomal dominant tubulointerstitial kidney disease-UMOD: a short review

**DOI:** 10.1186/s13023-025-03951-6

**Published:** 2025-08-06

**Authors:** Panpan Qiao, Zhaohui Wang, Jingyuan Xie

**Affiliations:** 1 Department of Nephrology, School of Medicine, Ruijin Hospital, Shanghai Jiao Tong University, No.197 Ruijin Er Rd, Shanghai, 200025 China; 2 Institute of Nephrology, School of Medicine, Shanghai Jiao Tong University, Shanghai, China

**Keywords:** ADTKD-UMOD, Hereditary kidney disease, *UMOD* gene, Renal dysfunction, Endoplasmic reticulum stress, Unfolded protein response

## Abstract

ADTKD-UMOD (Autosomal Dominant Tubulointerstitial Kidney Disease - Uromodulin) is a hereditary kidney disease caused by mutations in the *UMOD* gene, primarily characterized by renal dysfunction and related symptoms. This review aims to explore the clinical characteristics and molecular mechanisms associated with ADTKD-UMOD, highlighting the importance of understanding this condition for improved patient management. We will analyze the latest findings in the field of ADTKD-UMOD research, addressing its etiology, pathogenesis, clinical manifestations, and potential therapeutic strategies. Current research has identified various genetic mutations and their implications, yet challenges remain in fully elucidating the precise mechanisms by which these mutations lead to renal impairment. By synthesizing existing literature and addressing gaps in knowledge, this review seeks to enhance understanding of ADTKD-UMOD and promote effective clinical approaches to management and treatment.

## Introduction

Autosomal Dominant Tubulointerstitial Kidney Disease (ADTKD) is a group of disorders primarily involving damage to the renal tubuleinterstitium. Clinically, it is characterized by progressive renal insufficiency, mild proteinuria, association with pathogenic gene mutations, familial clustering, and a genetic predisposition, with no significant specific features in renal histopathology. Since the discovery of ADTKD, researchers have gradually recognized its complex genetic basis. The main pathogenic genes associated with ADTKD include *UMOD*, *HNF1B*, *MUC1*, *REN* and *SEC61A1* [1–5]. Mutations in these genes are closely linked to the development of the disease.

In recent years, with genetic testing becomes more and more widely used in clinical practice, the clinical identification rate of ADTKD has significantly improved. ADTKD is now recognized as one of the common monogenic diseases leading to chronic kidney disease, accounting for approximately 5% of all monogenic kidney disorders [6]. Significant progress has also been made in the screening of pathogenic genes associated with ADTKD. UMOD has been identified as the primary causative gene in ADTKD, associated with the ADTKD-UMOD subtype, representing 17%-61% of pathogenic variants across screening studies [3, 7–12].

ADTKD-UMOD is primarily characterized by tubulointerstitial fibrosis and progressive loss of renal function, typically manifesting as chronic kidney disease in early adulthood, accompanied by clinical features such as hyperuricemia and gout, and may eventually progress to end-stage kidney disease(ESKD) [13].

Recent studies have shown that the pathological features of ADTKD-UMOD include tubular atrophy, interstitial fibrosis, and electron-dense storage deposits within renal tubular epithelial cells [13]. These features are observed in renal biopsies as abnormal accumulation of UMOD protein, particularly in the endoplasmic reticulum. Studies have demonstrated a strong positive correlation (Spearman’s ρ = 0.709) between detection rates of abnormal UMOD accumulations and estimated glomerular filtration rate (eGFR) values [14].

The diagnosis of ADTKD has historically been challenging, primarily due to its atypical clinical features and the oversight of family history. However, with advancements in genetic screening technologies, particularly the application of next-generation sequencing (NGS), the genetic diagnosis of ADTKD has become more efficient and accurate [15]. As a result, an increasing number of ADTKD cases have been identified, and *UMOD* mutations have been confirmed as a significant cause of the disease [2]. Through in-depth research on the *UMOD* gene, scientists have not only uncovered its role in renal pathology but also provided potential targets for developing new treatment strategies. Future research will focus on how to improve the prognosis of ADTKD-UMOD patients and reduce its impact on quality of life through gene therapy or other interventions.

### Pathogenesis of ADTKD-UMOD

#### Function of the *UMOD* gene and mutation types

**Fig. 1:**
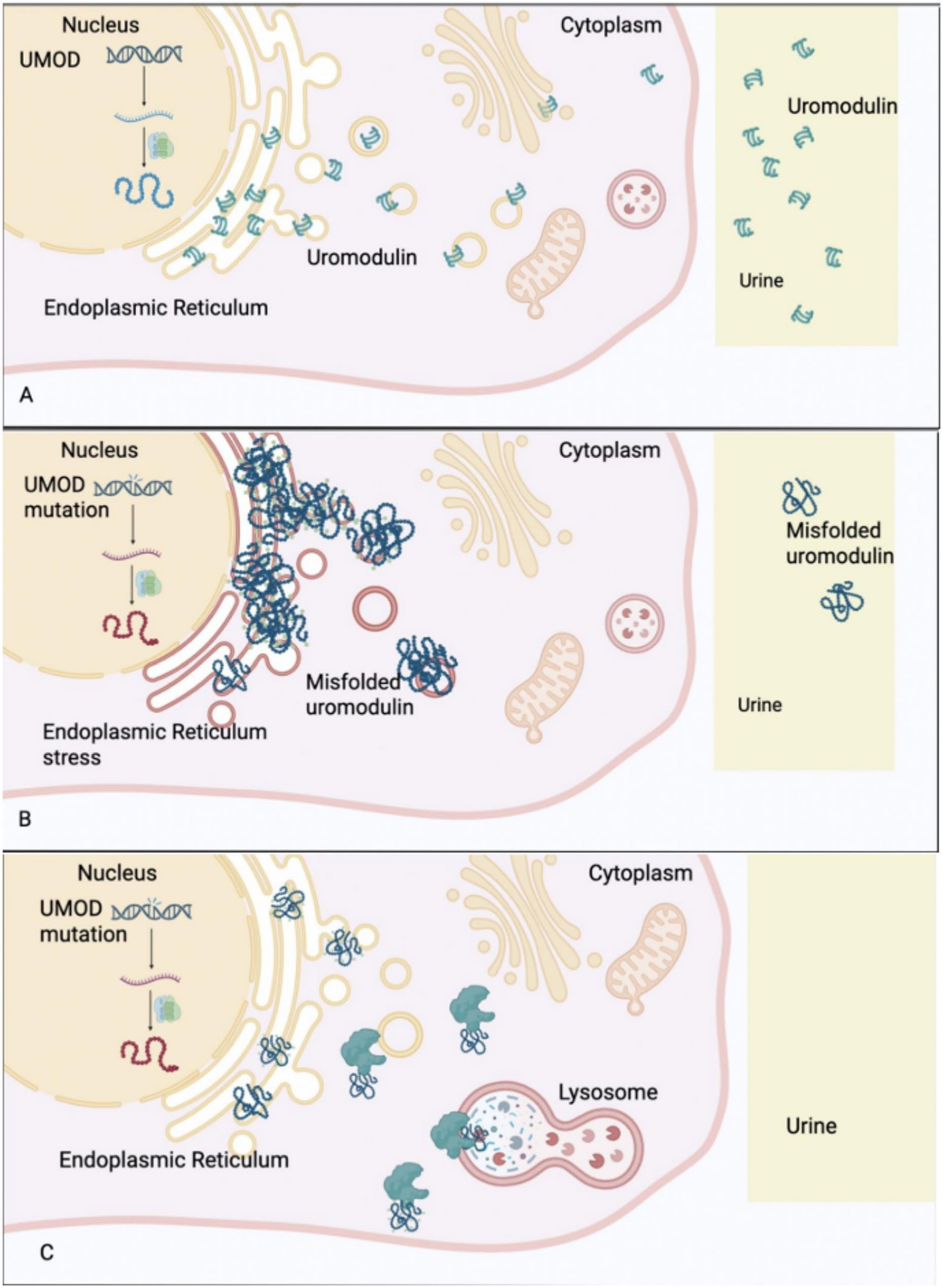
Expression of wild-type and mutant *UMOD. ***A**. The wild-type UMOD gene is translated into uromodulin, which is folded in the endoplasmic reticulum and secreted into the urine, becoming the most abundant protein in the urine of healthy individuals. **B**. UMOD gene mutations cause abnormal protein folding and deposition in the endoplasmic reticulum, triggering endoplasmic reticulum stress. **C**. Small molecules such as BRD4780 and therapeutic proteins like MANF can clear mutant UMOD abnormal proteins. [Created in https://BioRender.com]

The *UMOD* gene is located on chromosome 16p12.3 and consists of 11 exons, including one leader peptide (L), four epidermal growth factor (EGF I-IV)-like domains (among which EGF-II and EGF-III are predicted to be Ca2+-binding domains), a cysteine-rich domain of unknown function (D8C), and a C-terminal zona pellucida (ZP) domain with elastase resistance. It encodes the most abundant protein in normal urine, known as uromodulin or Tamm-Horsfall protein (THP). Uromodulin is produced by the thick ascending limb epithelial cells of the renal tubules and is released into the tubular lumen after regulation by serine proteases [Fig. 1A]. The primary functions of UMOD include regulating ion balance in renal tubules, participating in urine concentration, and protecting renal tubular epithelial cells from damage. UMOD promotes calcium reabsorption via the calcium channel protein TRPV5 in renal tubular epithelial cells, consequently inhibiting renal calculi formation [16, 17]. Additionally, UMOD exhibits antibacterial properties, protecting the kidneys from urinary tract infections [18, 19]. In 2009, Williams et al. confirmed that *UMOD* mutations occur in exons 3, 4, 5, and 7. Since then, an increasing number of studies have found that *UMOD* gene mutations are predominantly concentrated in exons 3 and 4 (> 95%). To date, over 100 pathogenic mutations have been identified, including missense, deletion, and indel mutations, missense mutations being the most common type [3, 7]. Interestingly, a pathogenic nonsense mutation in UMOD (e.g., c.1042 C > T p.(Gln348*)) was identified in a patient with congenital anomalies of the kidney and urinary tract (CAKUT) [20]. Mutations in the *UMOD* gene can cause ADTKD, while common polymorphisms in this gene are also associated with multiple disorders such as chronic kidney disease (CKD), hypertension, and cardiovascular diseases [18]. Studies utilizing large-population cohorts and ADTKD cohorts have identified candidate intermediate-effect *UMOD* variants. Some of these variants exhibit incomplete penetrance but demonstrate high genetic load in familial clusters of CKD [21].

#### Mechanisms of *UMOD* gene mutations and renal tissue damage

##### Impact on renal tubular epithelial cells

*UMOD* gene missense mutations often affect the formation of disulfide bonds between cysteine residues, thereby disrupting normal protein folding. This leads to abnormal accumulation of the protein in the endoplasmic reticulum (ER) of renal tubular epithelial cells, triggering endoplasmic reticulum stress (ER stress), activating cytotoxic responses and subsequent cellular apoptosis [Fig. 1B]. These changes manifest as swelling, deformation, and apoptosis of renal tubular epithelial cells, accompanied by dilation of the tubular lumen. Such degeneration not only compromises the structural integrity of the renal tubules but also significantly impairs their function, affecting the transport and metabolism of ions such as sodium, potassium, and calcium, and resulting in metabolic disturbances. For example, *UMOD* mutations are closely associated with hyperuricemia and gout, which arise due to reduced renal tubular excretion of uric acid [13].

##### Impact on tubulointerstitial immune responses

*UMOD* gene mutations also affect the immune responses in the renal tubulointerstitium, leading to chronic inflammation and fibrosis. Studies have shown that *UMOD* mutations can activate the immune system, resulting in the formation of anti-UMOD antibodies and the deposition of immune complexes within the renal tubules, thereby inducing localized inflammatory responses [22]. This immune response not only exacerbates renal tubular damage but may also promote tubulointerstitial fibrosis, ultimately leading to further loss of renal function and progression to ESKD [23, 24].

### Clinical management and diagnosis of ADTKD-UMOD

#### Clinical symptoms

ADTKD-UMOD is a rare genetic disorder caused by mutations in the *UMOD* gene. Its clinical manifestations are diverse, with patients typically presenting symptoms during adolescence or early adulthood. These symptoms include progressively worsening renal impairment, bland urinary sediment with minimal proteinuria, possible presence of uric acid crystals in the urine, elevated uric acid levels often accompanied by gout, and gradual progression to chronic renal failure. Additionally, some patients may experience urinary tract infections, which are related to the protective role of UMOD in urinary tract defense. ADTKD-UMOD follows an autosomal dominant inheritance pattern, meaning that a single copy of the mutated gene inherited from an affected parent can lead to the disease in offspring.

#### Imaging features

In imaging studies, the kidneys of ADTKD-UMOD patients typically appear normal or small, without significant renal cysts or other structural abnormalities, though mild renal atrophy may be observed. Renal biopsy results often reveal tubular atrophy and interstitial fibrosis, although these changes are not always evident on imaging. Studies have demonstrated that abnormal UMOD accumulation can be detected via PAS staining in up to 85% of ADTKD-UMOD patients [14].

#### Advances in the diagnosis of ADTKD-UMOD

In 2015, the KDIGO guidelines proposed definitive and provisional diagnostic criteria for ADTKD-UMOD [25]. The diagnostic criteria for ADTKD involve two key approaches: suspicion and definitive diagnosis. For suspected cases, either a family history of autosomal dominant CKD with characteristic clinical features (including inheritance pattern, progressive renal function decline, bland urinary sediment, minimal proteinuria, absence of early severe hypertension, no nephrotoxic drug exposure, normal/small kidneys on imaging, and childhood nocturia/enuresis) must be present, or in absence of family history, compatible renal biopsy findings (showing interstitial fibrosis, tubular atrophy, thickened/laminated basement membranes, potential tubular dilation/microcysts, and negative immunoglobulin/complement staining) or early-onset hyperuricemia/gout history are required. Definitive diagnosis necessitates either documented family history with both clinical and histological confirmation in at least one affected relative (noting biopsy alone is insufficient) or identification of pathogenic mutations in *UMOD* in the proband or affected family members, with all criteria maintaining strict clinicopathological and genetic correlation for accurate diagnosis [25].

### Treatment of ADTKD-UMOD

#### Basic treatments

ADTKD- UMOD currently has no specific treatment, and management primarily focuses on slowing disease progression and managing complications. The main treatment strategies remain based on the KDIGO guidelines for CKD [26]. However, there is currently no available data regarding the potential benefits of using angiotensin-converting enzyme inhibitors (ACEIs) or angiotensin receptor blockers (ARBs) in slowing the progression of chronic kidney disease in ADTKD patients. Patients with hyperuricemia can be treated with allopurinol or febuxostat (when allopurinol is not tolerated). It remains unclear whether allopurinol can slow the progression of kidney disease. In rare cases, patients may develop severe allergic reactions to allopurinol, and the medication should be discontinued immediately if a rash occurs. Additionally, allopurinol should be stopped before pregnancy, as it has been associated with cleft palate and other facial anomalies. For hyperuricemic patients with *UMOD* mutations, a strict low-purine diet has not been proven to be beneficial, and ADTKD patients should avoid the use of nonsteroidal anti-inflammatory drugs (NSAIDs) [25].

#### Disease progression and prognosis evaluation

The progression of ADTKD-UMOD is typically slow, with many patients developing ESKD between the ages of 20 and 70, making early identification and intervention particularly important [27]. Studies have shown that the prognosis of ADTKD-UMOD patients is influenced by various factors, including the type of mutation, the patient’s gender, and family history. For example, male patients generally face a higher risk of ESKD, while certain specific *UMOD* gene mutations may be associated with a more rapid decline in renal function. Patients with *UMOD* mutations involving cysteine substitutions tend to have a better prognosis [12]. With advancements in genetic testing technology, early identification of high-risk individuals carrying *UMOD* mutations can facilitate early intervention and family screening. Regular monitoring of renal function and uric acid levels can help improve long-term outcomes for patients.

#### Renal replacement therapy

When ADTKD patients progress to ESKD, kidney transplantation is an excellent option, as the genetic mechanism of ADTKD does not cause damage to the transplanted kidney [2, 28], and ADTKD patients can also choose dialysis (including hemodialysis or peritoneal dialysis).

#### Genetic counseling and prenatal/parental planning

All blood relatives of ADTKD patients should undergo genetic counseling. ADTKD-UMOD follows an autosomal dominant inheritance pattern, meaning individuals carrying *UMOD* gene mutations have a 50% chance of passing the mutation to their offspring. Currently, several technologies are available to assist patients with monogenic disorders in achieving better prenatal and parental planning. Non-invasive prenatal testing (NIPT) can analyze cell-free DNA (cfDNA) from maternal peripheral blood, which includes both maternal and fetal DNA. Using high-throughput sequencing technologies such as next-generation sequencing (NGS), NIPT can detect chromosomal abnormalities and certain monogenic disorders in the fetus, enabling early diagnosis [29]. Additionally, preimplantation genetic testing for monogenic diseases (PGT-M) has been successfully used to block the inheritance of pathogenic genes in patients with autosomal dominant polycystic kidney disease (ADPKD), offering hope for patients with monogenic inherited kidney diseases [30].

#### Latest therapeutic advances

Gene editing technologies, particularly CRISPR/Cas9, have brought unprecedented opportunities for the treatment of ADTKD-UMOD. For example, researchers have used CRISPR/Cas9 to create an ADTKD-UMOD mouse model and discovered that an endoplasmic reticulum chaperone protein, immunoglobulin heavy chain-binding protein (BiP), is upregulated during the progression of ADTKD-UMOD. They developed an ultra-sensitive plasmon-enhanced fluorescent-linked immunosorbent assay (p-FLISA), demonstrating that secretory immunoglobulin heavy chain-binding protein serves as a biomarker for early endoplasmic reticulum stress in the urine of ADTKD-UMOD patients [31]. Subsequently, researchers, by monitoring BiP, discovered a secreted endoplasmic reticulum protein called mesencephalic astrocyte-derived neurotrophic factor (MANF). Overexpression of this protein was found to promote autophagy, clear mutant UMOD in mouse models, and thereby protect kidney function [32]. In 2024, researchers have investigated the molecular mechanisms responsible for the processing of mutant UMOD both in vitro and in vivo. They discovered that TMED2, TMED9, and TMED10 interact with UMOD and regulate its trafficking. A small molecule targeting TMED (BRD4780) promoted the intracellular retention and clearance of mutant UMOD, thereby improving the disease phenotype [33](Fig. 1C).

## Conclusion

With the improved understanding of ADTKD, multiple global genetic screening studies have provided more precise epidemiological data, while in-depth research on disease-causing mouse models and targeted protein clearance research has expanded the therapeutic exploration. In conclusion, the landscape of ADTKD-UMOD research is evolving, with the promise of new discoveries on the horizon.

## Data Availability

Not applicable.
